# How preceptors develop trust in continuity clinic residents and how trust influences supervision: A qualitative study

**DOI:** 10.1007/s40037-021-00694-5

**Published:** 2021-12-16

**Authors:** John C. Penner, Karen E. Hauer, Katherine A. Julian, Leslie Sheu

**Affiliations:** Department of Medicine, School of Medicine, University of California, San Francisco, USA

**Keywords:** Trust, Supervision, Continuity clinic, Graduate medical education

## Abstract

**Introduction:**

To advance in their clinical roles, residents must earn supervisors’ trust. Research on supervisor trust in the inpatient setting has identified learner, supervisor, relationship, context, and task factors that influence trust. However, trust in the continuity clinic setting, where resident roles, relationships, and context differ, is not well understood. We aimed to explore how preceptors in the continuity clinic setting develop trust in internal medicine residents and how trust influences supervision.

**Methods:**

In this qualitative study, we conducted semi-structured interviews with faculty preceptors from two continuity clinic sites in an internal medicine residency program at an urban academic medical center in the United States from August 2018–June 2020. We analyzed transcripts using thematic analysis with sensitizing concepts related to the theoretical framework of the five factors of trust.

**Results:**

Sixteen preceptors participated. We identified four key drivers of trust and supervision in the continuity clinic setting: 1) longitudinal resident-preceptor-patient relationships, 2) direct observations of continuity clinic skills, 3) resident attitude towards their primary care physician role, and 4) challenging context and task factors influencing supervision. Preceptors shared challenges to determining trust stemming from incomplete knowledge about patients and limited opportunities to directly observe and supervise between-visit care.

**Discussion:**

The continuity clinic setting offers unique supports and challenges to trust development and trust-supervision alignment. Maximizing resident-preceptor-patient continuity, promoting direct observation, and improving preceptor supervision of residents’ provision of between-visit care may improve resident continuity clinic learning and patient care.

**Supplementary Information:**

The online version of this article (10.1007/s40037-021-00694-5) contains supplementary material, which is available to authorized users.

## Introduction

Supervision of resident trainees requires balance between ensuring safe, high-quality patient care and enabling learners to operate at the edge of their developmental level [1–4] Working with less supervision at the threshold of one’s competence can maximize learner growth [4–6]. To advance in their clinical roles, resident learners must earn their supervisors’ trust. Therefore, trust, defined as “having faith or confidence in someone or something,” [7] is a growing area of interest in medical education.

Medical education literature identifies five main factors influencing supervisors’ trust in trainees in the clinical setting: trainee, supervisor, trainee-supervisor relationship, context, and task [8, 9]. Observations of *trainees*, including their clinical care, communication, and reliability, promote the development or erosion of trust. *Supervisor* characteristics, such as their innate tendency to trust trainees and their confidence in their own clinical and supervisory skills, also affect trust [1–3, 8–11]. *Relationship* factors, including longitudinal trainee-supervisor interactions, promote trust [1–3, 8–12]. *Contexts* with high-acuity situations and high-complexity *tasks* engender higher thresholds for trust [1–3, 8–12]. Barriers to and accelerators of trust formation relate to the five factors and influence the evolution of resident supervision throughout training [2].

Studies investigating trust have focused on inpatient settings [1–3, 8, 10, 11, 13]. However, the continuity clinic setting comprises an important part of training in internal medicine and other specialties [14, 15]. The workflow in continuity clinics suggests that factors contributing to trust formation may manifest differently than in inpatient settings. In the United States (US), the required continuity clinic setting often affords trainees longitudinal experience over several years with the same patients and supervisors [16]. Additionally, a substantial amount of this patient care occurs between office visits, where the supervisor is not immediately present [17–20].

In the US, the Accreditation Council for Graduate Medical Education requires that all residency programs provide “progressive responsibility for patient management and graded supervision” [14]. As educators and accreditation bodies emphasize the need for improved continuity clinic education [14, 15], understanding how supervisors develop trust in residents in these settings can inform teaching practices and systems designs that promote accurate trust assessments, facilitate appropriate supervision, and support resident knowledge, skill acquisition, and readiness for unsupervised practice [2, 9]. This study aims to use the theoretical framework of the five factors that influence trust to explore how preceptors in continuity clinics develop trust in residents and how trust influences supervision [9].

## Methods

This qualitative study uses an interpretivist paradigm. We conducted semi-structured interviews with continuity clinic preceptors in an internal medicine residency program. We used thematic analysis with sensitizing concepts related to the five factors of trust to explore preceptors’ experiences determining trust while supervising residents [21–23]. The UCSF Institutional Review Board deemed the study exempt from review.

This study was carried out at two continuity clinic sites within the internal medicine residency program at the University of California, San Francisco (UCSF), an urban, quaternary care center. Residents complete approximately 150 continuity clinic sessions (half-days) over three years, with a panel of approximately 130 patients per resident. First-year residents have two dedicated outpatient months with three sessions a week of continuity clinic, seeing up to four patients a session. For the remaining months, they have up to two continuity clinic sessions a month. Second- and third-year residents alternate inpatient and outpatient months. During outpatient months, they have three sessions of continuity clinic a week, seeing up to six patients a session. They provide care between patient visits by responding to patient messages, reviewing and acting upon results, reviewing consultant notes, and coordinating care.

Preceptors are faculty general internal medicine physicians assigned to a given half-day for continuity with residents. All serve as longitudinal preceptors to residents for at least one, if not all three years of residency. Preceptor-resident ratios are one preceptor to 2–3 residents. During precepting encounters, residents independently see a patient and present the history, exam, assessment, and plan to a preceptor who then evaluates the patient with the resident. The resident discusses the care plan with the patient and closes the visit. Residents are assigned a longitudinal preceptor but may precept with multiple preceptors in a session. Preceptors serving as the longitudinal preceptor for residents are available to answer questions and assist with between-visit tasks throughout residency.

Preceptors with at least one year’s experience precepting at least one half-day per week within two clinic sites were invited to participate. JCP invited all preceptors (*n* = 37) by email to participate in interviews between August 2018 and June 2020, with up to two email reminders. JCP completed all the interviews. We conducted analysis concurrent with data collection and ceased scheduling interviews when we stopped identifying new themes during our data review [24].

We designed the interview guide to address preceptor practices related to trust and supervision of continuity clinic residents, with a focus on exploring the five factors of trust (Appendix A of the Electronic Supplementary Material) [9]. JCP piloted the interview guide with two preceptors. JCP and LS reviewed pilot interview transcripts and revised the guide for clarity and flow. These pilots were included in the final dataset as changes to the guide did not alter interview content. All interviews were professionally transcribed and deidentified.

We analyzed interviews using thematic analysis [21, 22]. We generated a codebook with subcodes based on recurring ideas we identified in the data. The codebook was informed by sensitizing concepts related to the five factors of trust [9]. Investigators remained open to other ideas outside of the five factors [23]. JCP and LS reviewed two initial transcripts to develop a preliminary codebook; two others (KEH, KAJ) coded another transcript with JCP and made further codebook refinements after reconciliation and discussion. Investigators made minor codebook revisions during additional transcript coding. All transcripts were double-coded by JCP and one other investigator (KEH, KAJ, LS). Discrepancies were reconciled through discussion.

Codes were organized using Dedoose analytic software v. 8.3.11 (Sociocultural Research Consultants, LLC, Los Angeles, California). All investigators independently reviewed coded excerpts to identify themes, which we discussed and refined through group discussion. After 16 interviews, we agreed we had gathered a wide range of conceptually nuanced data without hearing new ideas and thus stopped scheduling interviews [24].

We reflected on how the team’s composition brought different perspectives to the study [25]. At the time of the study, JCP was an internal medicine resident who experienced preceptors’ trust and supervision, allowing him to interpret data from the learner perspective. KEH was an internist, former preceptor, medical education researcher, and expert on trust. She brought perspectives external to the resident continuity clinic context to the interpretation of data. KAJ was program director of the UCSF Primary Care Residency track and an experienced preceptor, with an intimate understanding of the resident continuity clinic experience. LS was an assistant professor and faculty preceptor in the resident continuity clinic, who has conducted prior trust research. We shared our perspectives on the data via email exchanges and group meetings. We also discussed how JCP’s role as a resident could have influenced preceptor responses by making them hesitant to disclose certain supervisory practices or share stories that might identify one of his peers, though interviewees spoke in-depth and seemingly freely about a range of positive and challenging precepting experiences.

To promote trustworthiness of our results, we implemented member checking with four participants [26]. We shared a synthesis of preliminary results and solicited reactions via email. All four endorsed the findings. Three offered reflections that we incorporated into the analysis, such as refining the role of directly observing residents communicating with patients.

## Results

Eighteen preceptors responded, and 16 (43%) participated. Eleven participants (69%) were women. Six (37.5%) were assistant professors, four (25%) associate, and six (37.5%) full professors. Preceptors had been on faculty for 2–28 years (average 11.4) and precepted 1–3 sessions a week (average 1.9). Interviews lasted 30–62 min (average 47.0).

**Fig. 1:**
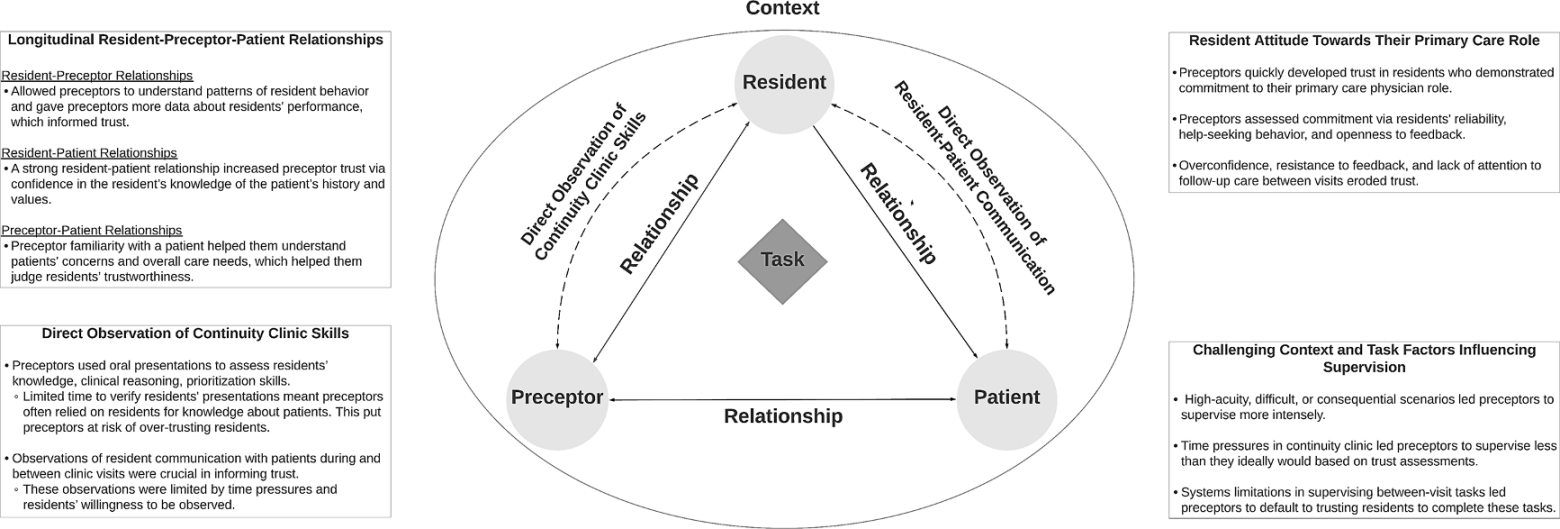
A summary of the factors that influence preceptor trust in residents and how trust relates to supervision in the continuity clinic setting

We identified four key drivers of trust development and supervision in the continuity clinic setting: 1) longitudinal resident-preceptor-patient relationships 2) direct observations of continuity clinic skills, 3) resident attitude towards their primary care physician role, and 4) challenging context and task factors. These themes and related subthemes are elaborated below and depicted in Fig. 1. Representative quotations include participant numbers in parentheses.

### Longitudinal resident-preceptor-patient relationships

Preceptors regarded longitudinal relationships between resident and preceptor, resident and patient, and preceptor and patient as invaluable in informing trust.

Observing and interacting with residents over time allowed preceptors to understand patterns of resident behavior, including their preparation, thoroughness, clinical reasoning, patient communication, and follow-through: “*It’s the touchpoints, how many times I precept with that person […] that lets me see how they work and the level of trust I have*” [4]. Resident-preceptor relationships that developed over time gave preceptors more data about a resident’s performance, enhancing their comfort discussing feedback: “*It’s easier to give feedback when you’ve worked with the resident over a long time because you have a higher number of interactions*” [2]. Longitudinal resident-preceptor relationships also allowed preceptors to observe for feedback implementation, enabling residents to earn more trust.

In the absence of a longitudinal relationship with a resident, preceptors tended rely on the resident’s training level to determine trust: “*Simply by virtue of having gotten to that level of experience, even if they haven’t worked with me directly, I’m willing to extend trust to a [third year resident] I’ve never worked with*” [6]. Most preceptors started with a high baseline level of trust, including with first year residents, until they saw a reason to trust less. Preceptors also valued relationships with residents outside the continuity clinic setting, such as through mentoring, inpatient teams, or clinical conferences, to guide trust. Occasionally, when preceptors noted concerns about a resident’s trustworthiness, they discussed their impressions with other preceptors to confirm or refute their concerns and brainstorm ways to help the resident.

When residents had seen a patient multiple times, preceptors trusted them to have a deep understanding of that patient’s medical history and personal values. Preceptors identified the resident-patient continuity clinic relationship as particularly important to motivate patient behavior change and increase engagement in care: “*Another [important factor in trust], probably more so in an outpatient setting than the inpatient setting, is feeling there’s a rapport, that the patient feels a connection to the [resident] and is willing to follow through.*” [14].

Preceptor familiarity with a patient provided more information to inform trust assessments: “*When a resident sees my patients, I know them inside-out, so I know immediately if they’re not picking up on something [or] didn’t appreciate some important part of the history”* [6]. Preceptors felt better able to understand patients’ concerns or the severity of their current presentation, which helped them judge residents’ performance to inform trust.

### Direct observations of continuity clinic skills

Preceptors valued observations of residents’ oral presentations and communication with patients as providing key information to determine their level of trust. They also acknowledged unique limitations to these observations in continuity clinics.

During residents’ oral presentations, preceptors assessed residents’ knowledge, clinical reasoning, and ability to prioritize and manage multiple patient problems. Preceptors recognized the challenge of prioritizing patient problems in continuity clinics, as patients often had multiple acute concerns and chronic diseases. Preceptors tended to seek evidence of organization of patient problems and management plans to infer thoroughness: “*There’s a level of entrustment that has to do with their prioritization of the issues. I think this is a little different than inpatient medicine […] there are several problems […] If I’m getting the sense that there is an item that maybe was addressed, but in a superficial way, but it’s really important; then I start getting a little bit nervous ”* [7].

Though oral presentations were crucial in informing trust, many preceptors also acknowledged limitations. They often relied on residents for knowledge about patients, with limited time to verify information by repeating parts of the patient interview or reviewing chart notes. Preceptors therefore felt unable to know what was omitted, and thus risked over-trusting the resident: “*You could have a resident who presents really well but is glossing over things. Maybe we would assume that because they’re able to present well, they have a really good ability to formulate what’s going on with the patient. It’s not always the case*” [12]. Preceptors rarely verified residents’ orders placed after an encounter or received follow-up on patient outcomes, making it difficult to ascertain residents’ execution of care plans. With these limitations, preceptors tended to default to trusting residents’ abilities to carry out agreed-upon plans.

Observations of resident communication with patients during and, uncommonly, between clinic visits were crucial in informing trust. Preceptors found resident rapport with patients particularly important in the continuity clinic setting; they perceived strong resident-patient rapport enhanced patients’ understanding of their diagnoses and willingness to follow through with plans. However, these observations were limited by time pressures and residents’ willingness to be observed. “*The amount of time I spend in the room [observing] largely has to do with the other pieces of my role. I just don’t have that luxury of being in the room for long periods of time, because I have other people I’m responsible for*” [7]. Another facilitator shared: “*[Residents are] very worried about being on time, and I’m sure that when I go in the room they feel like they have to do something different […] But I always see things in the room that surprise me. I think we should do it more.”* [12].

Between clinic appointments, preceptors’ observations of residents responding to patient messages or communicating results to patients in a timely and patient-centered manner via the electronic health record increased trust. However, preceptors found their ability to observe when and how residents follow-up with patients to be intermittent and serendipitous.

### Resident attitude towards their primary care physician role

Residents who conveyed commitment to their primary care physician role quickly engendered trust. They were thorough and reliable in patient care tasks, seemed eager to grow and learn, and regularly sought help and welcomed feedback. Conversely, residents who seemed overconfident, inattentive to follow-up between visits, or resistant to feedback signaled disinterest in continuity clinic, leaving preceptors concerned that the resident was not invested in delivering highest quality care: *“If I have the sense that the resident is really rushing to try to get out of there, that makes me feel a little bit of a level of distrust; this person doesn’t really want to be in primary care clinic.” *[14].

### Challenging context and task factors influencing supervision

High-acuity, consequential, or challenging clinical scenarios seemed to require intense supervision regardless of trust in a resident. However, the context of a busy clinic day could lead preceptors to supervise less, even if that reality did not align with their level of trust: “*If someone is running really behind there probably isn’t a lot of time to [go in the room with the resident] […] If I’m covering a lot of people for precepting […] there might be less time for me to actually be in the room even if I thought it would be a good idea.*” [5].

With regard to tasks, preceptors expressed concerns about systems’ limitations that prevented them from supervising between-visit tasks and risked trust-supervision misalignment: “*We don’t have great systems for doing that […] Let’s say we planned that the resident is going to check-in with the patient after a week on how they’re doing with a new therapy […] I’ve probably forgotten all about it.*” [6].

Preceptors also highlighted that outpatients may not complete their tests, procedures, or follow-up for months, and the resident may work with a different preceptor during the patient’s follow-up visit. These challenges left preceptors feeling resigned to providing minimal supervision for between-visit tasks. They also noted that they rarely saw how residents handled patient results, messages, or phone calls. Preceptors shared that they occasionally flagged patients for follow-up, particularly in cases of less trust in the resident, high patient acuity, or diagnostic uncertainty. Otherwise, they largely trusted residents to manage these tasks and seek help when needed.

## Discussion

We aimed to understand how faculty preceptors develop trust in continuity clinic residents and how trust informs supervision. Our findings demonstrate the central role of longitudinal relationships, direct observation, resident attitudes, and context- and task-specific factors in trust formation and supervision in resident continuity clinics.

Longitudinal resident-preceptor-patient relationships were vital to trust formation. Studies on trust highlight resident-preceptor continuity for promoting accurate trust assessments and facilitating developmentally appropriate, progressive participation in clinical care [9, 27–29]. In our U.S. study, where the Accreditation Council for Graduate Medical Education requires residency continuity clinics for internal medicine trainees [14], longitudinal relationships over years allowed preceptors to identify patterns of resident behavior, residents’ attitudes towards their primary care physician role, and opportunities to provide residents feedback. Though attitude has been identified as an information source contributing to supervisors’ trust [30], preceptors’ inferences about trustworthiness based specifically on residents’ investment in their continuity patients and desire to improve is unique. This focus may reflect the protracted timeline of care for patients in the continuity clinic setting that requires consistent, longitudinal follow-up and proactive help-seeking behaviors, unlike inpatient or procedural contexts where care is relatively time bound. Based on our findings, we recommend medical educators prioritize resident-preceptor continuity in designing resident continuity clinics to promote trust development.

Although our study was conducted within a medical education system that emphasizes educational continuity [14, 29], preceptors acknowledged challenges with fostering effective longitudinal relationships due to schedule variability for residents and preceptors. Prior studies highlight the prevalence of discontinuity in medical training and the need to create curricular structures to mitigate this [31, 32]. In the absence of longitudinal relationships, some preceptors described corroborating trust assessments with other preceptors. Formalizing such practices for preceptors to discuss learners’ progress may help offset the challenges of limited resident-preceptor continuity, and thereby enhance teaching and supervision [33, 34]. Our study also highlighted that longitudinal relationships do not always yield trust development. These instances, while uncommon, can lead to dissatisfaction within the preceptor-resident relationship and negatively impact resident engagement and career choice [35, 36]. Faculty development, particularly around skills to address barriers to trust, provide constructive feedback, and facilitate relationship-building could promote productive and meaningful longitudinal relationships within the continuity clinic setting.

Beyond the resident-preceptor relationship, preceptors also valued resident-patient relationships in determining trust. This finding aligns with broader literature on the importance of provider-patient relationships in continuity clinic settings[37, 38] and is not emphasized in prior research on trust in inpatient and procedural settings [1–3, 11, 39]. While the resident-patient relationship was important, preceptors acknowledged limited opportunities for direct observation of resident-patient communication for a variety of reasons, including limited time. This challenge echoes prior literature on continuity clinic supervision and underscores the previously described need for preceptor models that decrease time constraints and facilitate direct observation [20, 40]. Prior recommendations on supporting direct observation include discussing its utility with residents and implementing faculty development on observation and feedback [11, 41, 42]. These may be helpful tools for continuity clinic educators seeking to overcome barriers to direct observation.

Finally, the importance of preceptor continuity with residents’ patients highlights how the preceptor-patient relationship contributes differently to trust in the continuity clinic setting compared to other contexts. Continuity clinic preceptors often had less familiarity than residents with their patients, presenting a patient safety and educational challenge. Prior literature highlighted that over-trust, which can occur when supervisors have few experiences with a trainee or face high clinical demands, can lead to unsafe patient care [9, 30, 43]. In our study, preceptors feared they over-trusted residents due to their own limited knowledge of and continuity with residents’ patients. Systems that promote preceptor familiarity with residents’ patients, such as preferentially scheduling residents’ patients with longitudinal faculty preceptors when the resident is unavailable, may support preceptors in making accurate trust assessments without compromising teaching or patient care.

Trust and supervision of residents providing care between clinic visits, primarily through the electronic health record, was particularly challenging. Prior studies found variable resident completion of between-visit tasks and frequent deficiencies in patient-centered communication, with no studies on the quality of task completion or supervisor oversight [44–46]. Further research on resident performance and preceptor supervision of task completion, particularly related to patient communications and abnormal results, may reveal effective ways to support the alignment of trust and supervision in between-visit care and facilitate feedback and coaching that promote residents’ growth.

This study has limitations. Our participants came from a single institution; findings may not be transferable to other contexts. We did not corroborate preceptors’ perceptions by collecting residents’ perspectives on trust and supervision. Results are based on preceptor self-report and did not include observations.

In summary, we identified unique contributors and challenges to trust development and alignment of trust with supervision in the continuity clinic setting. To promote supervisors’ trust in residents, we encourage educators to think critically about strategies to maximize resident-preceptor-patient continuity, promote direct observation, and improve supervision of residents’ provision of between-visit care.

## Supplementary Information

Supplementary materials include the interview guide
