# Supplementary material for: How preceptors develop trust in continuity clinic residents and how trust influences supervision: A qualitative study
Source: Perspect Med Educ. 2021 Dec 16;11(2):73–9. doi: 10.1007/s40037-021-00694-5 (PMC8941004; doi:10.1007/s40037-021-00694-5)
Supplement: Supplementary file 1 — Supplementary materials include the interview guide [file 40037_2021_694_MOESM1_ESM.docx]

**Appendix A**:

**Interview guide.**

**Part 1: Understanding trust in clinic.**

1. To begin, I’d like to understand how you approach trust and supervision of residents in clinic.
   1. First, what does it mean to you to trust residents with patient care in clinic?
   2. What makes you decide if you trust a resident with certain tasks? Can you give an example?
   3. How does your level of trust influence how you supervise them during or between patient encounters?
      1. Probe: Can you give an example of what you do differently when you have more trust with a resident?
      2. Probe: Can you give an example of what you do differently when you don’t trust a resident?
   4. Generally speaking, how, if at all, does your trust and supervision change from intern to R3?”
   5. How does the need to encourage and develop autonomy impact how you supervise residents in the ambulatory setting?
   6. When you are serving as the backup/partner attending, do you think about trust? How does that influence your supervision??
2. Next, I’d like to talk about how trust fluctuates with individual residents over time.
   1. Think about a current or graduated R3 that you worked with longitudinally and got to know well.
      1. For this particular resident, what, facilitated your relationship?
      2. How does (or did) your relationship influence your trust and supervisory style over time?
      3. What resident behaviors beyond ones we may we have already discussed, influenced these changes?
   2. Now, think about a current or graduated R3 that you worked with but did not get to know well.
      1. What were the barriers to establishing a relationship with this resident?
      2. How does (or did) this influence your trust and supervisory style?
      3. Are there factors outside of your precepting relationship with this resident that influenced changes in trust or supervision?

**Part 2: Understanding specific supervisory practices in clinic.**

1. Now, I would like to ask about specific practices when precepting residents.
   1. When, if at all, are you choosing to directly supervise residents during the clinical encounter?
      1. If yes: During which portions do you find yourself doing it most and why? What impact does this have on the development of trust with residents?
      2. If no: What influences your decision not to?
   2. When, if at all, do you decide to reference the patient’s chart while precepting?
   3. When, if at all, do you decide to elicit additional history from a patient?
   4. When, if at all, do you decide to perform a physical exam on a patient?
   5. When do you decide to give residents feedback (reinforcing or constructive)?
   6. What other factors in a clinic session, outside of the precepting encounter itself, influence your supervisory style on any given day?
2. Next, I would like to ask about your supervision practices outside of the clinical encounter.
   1. I’d like to start by asking about your practices when attesting notes.
      1. When do you decide to review resident notes before attesting them?
      2. When do you decide to review the chart in greater detail before attesting?
   2. Now, I’d like to ask about your practices reviewing any follow-up or action items determined for a patient case you precepted.
      1. How do you decide when to follow-up on action items to make sure they are done? Can you give an example.
      2. How do you decide when to offer feedback on how residents handled these action items (reinforcing or constructive)?
   3. Now, I’d like to ask about your practices with overseeing resident inbaskets.
      1. How do you provide oversight for the work your resident(s) is/are conducting virtually?
      2. When do you decide to provide residents with feedback about management?
3. Next, I’d like to ask just a couple questions about how virtual precepting with COVID19 has impacted your trust and supervisory practices.
   1. How, if at all, has COVID19 and the transition to virtual precepting impacted how you assess or develop trust with residents?
   2. How has the transition to virtual precepting impacted how you supervise during the encounter?
   3. Have you noticed any effects of virtual precepting as it pertains to your assessing trust?
      1. Any benefits? Any limitations?
   4. How, if at all, has virtual precepting changed the way you review residents’ patient charts? What about follow-up items?

**Part 3: Reflections (Choose ones not answered already during the interview)**

1. The last few questions are reflective. How has the way you trust and supervise residents changed over time on faculty?
2. What do you see as unique advantages or benefits you have in the ambulatory setting to providing supervision and assessing trust?
3. What do you see as unique challenges to providing supervision and assessing trust in the ambulatory setting?
4. Is there anything else you would like to share about trust and supervision in the ambulatory setting?
